# Resurgence of Vaccine-Preventable Diseases in Venezuela as a Regional Public Health Threat in the Americas

**DOI:** 10.3201/eid2504.181305

**Published:** 2019-04

**Authors:** Alberto E. Paniz-Mondolfi, Adriana Tami, Maria E. Grillet, Marilianna Márquez, Juan Hernández-Villena, María A. Escalona-Rodríguez, Gabriela M. Blohm, Isis Mejías, Huníades Urbina-Medina, Alejandro Rísquez, Julio Castro, Ana Carvajal, Carlos Walter, María G. López, Philipp Schwabl, Luis Hernández-Castro, Michael A. Miles, Peter J. Hotez, John Lednicky, J. Glenn Morris, James Crainey, Sergio Luz, Juan D. Ramírez, Emilia Sordillo, Martin Llewellyn, Merari Canache, María Araque, José Oletta

**Affiliations:** Clínica IDB Cabudare, Instituto de Investigaciones Biomédicas IDB, Cabudare, Venezuela (A.E. Paniz-Mondolfi, M. Márquez, M.A. Escalona-Rodriguez, G.M. Blohm);; Venezuelan Science Incubator, Barquisimeto, Venezuela (A.E. Paniz-Mondolfi, M. Márquez, M.A. Escalona-Rodríguez, G.M. Blohm, I. Mejías);; University of Groningen, University Medical Center Groningen, Groningen, the Netherlands (A. Tami);; Facultad de Ciencias de la Salud, Universidad de Carabobo, Valencia, Venezuela (A. Tami);; Universidad Central de Venezuela, Caracas (M.E. Grillet, J. Hernández-Villena, A. Rísquez);; Universidad Centrooccidental Lisandro Alvarado, Barquisimeto (M. Márquez);; University of Florida, Gainesville, Florida, USA (G.M. Blohm, J. Lednicky, J.G. Morris Jr.);; Rotary International, Houston, Texas, USA (I. Mejías);; Sociedad Venezolana de Puericultura y Pediatría, Caracas (H. Urbina-Medina);; Sociedad Venezolana de Salud Pública/Red Defendamos la Epidemiología Nacional, Caracas (J. Castro, A. Carvajal, C. Walter, J. Oletta);; Sociedad Venezolana de Infectología, Caracas (M.G. López);; University of Glasgow, Glasgow, Scotland, UK (P. Schwabl, L. Hernández-Castro, M. Llewellyn);; London School of Hygiene and Tropical Medicine, London, UK (M.A. Miles);; Baylor College of Medicine National School of Tropical Medicine, Houston (P.J. Hotez);; Instituto Leônidas e Maria Deane/FIOCRUZ, Manaus, Brazil (J. Crainey, S. Luz);; Universidad del Rosario, Bogotá, Colombia (J.D. Ramírez);; Mount Sinai Saint Luke’s, New York, New York, USA. (E. Sordillo);; Hospital de Niños José Manuel de los Ríos, Caracas (M. Canache);; Universidad de Los Andes, Mérida, Venezuela (M. Araque)

**Keywords:** measles, diphtheria, polio, Venezuela, outbreak, vaccine-preventable diseases, vector-borne infections, viruses, Americas, vaccines, vaccination, immunization

## Abstract

Venezuela’s tumbling economy and authoritarian rule have precipitated an unprecedented humanitarian crisis. Hyperinflation rates now exceed 45,000%, and Venezuela’s health system is in free fall. The country is experiencing a massive exodus of biomedical scientists and qualified healthcare professionals. Reemergence of arthropod-borne and vaccine-preventable diseases has sparked serious epidemics that also affect neighboring countries. In this article, we discuss the ongoing epidemics of measles and diphtheria in Venezuela and their disproportionate impact on indigenous populations. We also discuss the potential for reemergence of poliomyelitis and conclude that action to halt the spread of vaccine-preventable diseases within Venezuela is a matter of urgency for the country and the region. We further provide specific recommendations for addressing this crisis.

Residents of Venezuela are struggling to survive in a country with the world’s highest annual inflation rate, 46,305% (*1*), and a minimum monthly wage of just USD $1.79 (5,196,000 Bolivars). The International Monetary Fund predicted an inflation rate of 1,000,000% by the end of 2018 (*1*). Sixty-six percent of the population lives in extreme poverty (*2*), amid escalating violence and a crumbling healthcare infrastructure more typical of conflict zones or war-torn nations. More than 280,000 children are at risk for death from severe malnutrition (*3*). According to the last official nationwide epidemiologic bulletin, published in 2016, infant and maternal mortality had risen by 30% and 65%, respectively, over the previous year (*2*); no further national epidemiologic records have been released.

Long-term shortages of essential medicines and medical supplies (only 30% of basic drugs to treat infectious diseases are available in public hospitals) (*2*), interruption of epidemiologic surveillance systems, weakening of immunization programs, and an unprecedented exodus of trained medical personnel have set the stage for the resurgence of vectorborne and vaccine-preventable infections (*4*–*6*). A striking manifestation is the ongoing malaria epidemic in Venezuela (*6*–*9*), now approaching half a million cases per year and continuing to increase at rates exceeding those previously reported anywhere in the world (*10*).

Recently, the return of measles and other vaccine-preventable childhood infections in Venezuela (Figure 1), as well as the potential for expansion of outbreaks beyond Venezuela’s borders, has been recognized by the World Health Organization (WHO) and the Pan American Health Organization (PAHO) (*9*). In Colombia alone, 25 cases of imported measles in migrants from Venezuela had been recorded as of October 2018 (*9*,*10*). The continued mass exodus of ≈2 million persons from Venezuela since 2014 (*9*), not only to Colombia (>820,000 migrants) but also to Ecuador (>450,000) and Brazil (>57,000) (*11*–*13*), represents an ongoing risk that vaccine-preventable diseases will be carried with them. The United Nations High Commissioner for Refugees (UNHCR) has launched a supplementary appeal for funding for these affected countries (*14*). Simultaneously, the spread of vaccine-preventable diseases must be tackled urgently within Venezuela, with an emphasis on containing outbreaks locally and at critical border areas.

**Figure 1:**
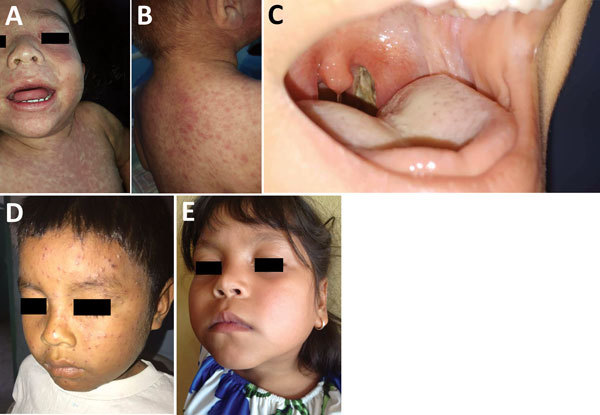
Clinical features observed in children infected with vaccine-preventable diseases, Venezuela, 2017–2018. A, B) Classic morbilliform measles rash in a Creole infant from Caracas. Note the pronounced erythematous confluent macules and patches on face and subsequently a cephalocaudal spread onto the trunk and extremities. C) Thick, gray membrane covering the pharynx and posterior aspects of tonsils in a case of diphtheria. D) A Pemón Amerindian child with a classical varicella rash exhibiting various lesion stages. E) Swelling of the parotid glands in a girl with mumps from the state of Lara in central-western Venezuela.

## Measles

In Venezuela, circulation of wild measles was interrupted in February 2007 after a mass vaccination campaign that followed outbreaks in 2001 and 2006 (*15*). However, since 2017, measles has reemerged in Venezuela, particularly within vulnerable indigenous populations, and has subsequently reached neighboring countries (Figure 1, panel A) (*16*). As of October 23, 2018, Venezuela had contributed 68% (5,525/8,091 cases) of the measles cases reported in the Americas and most of the measles-related deaths (73/85) (*16*). Genotyping of the measles virus isolated from patients from Venezuela (imported cases) in Brazil, Colombia, Ecuador, and Peru confirmed that the strains were genotype D8, lineage MVi/Hulu Langat.MYS/26.11 (*16*). The D8 genotype is associated with endemic transmission in Asia and the Pacific (*17*) and is the main lineage circulating currently in South America (*16*).

**Figure 2:**
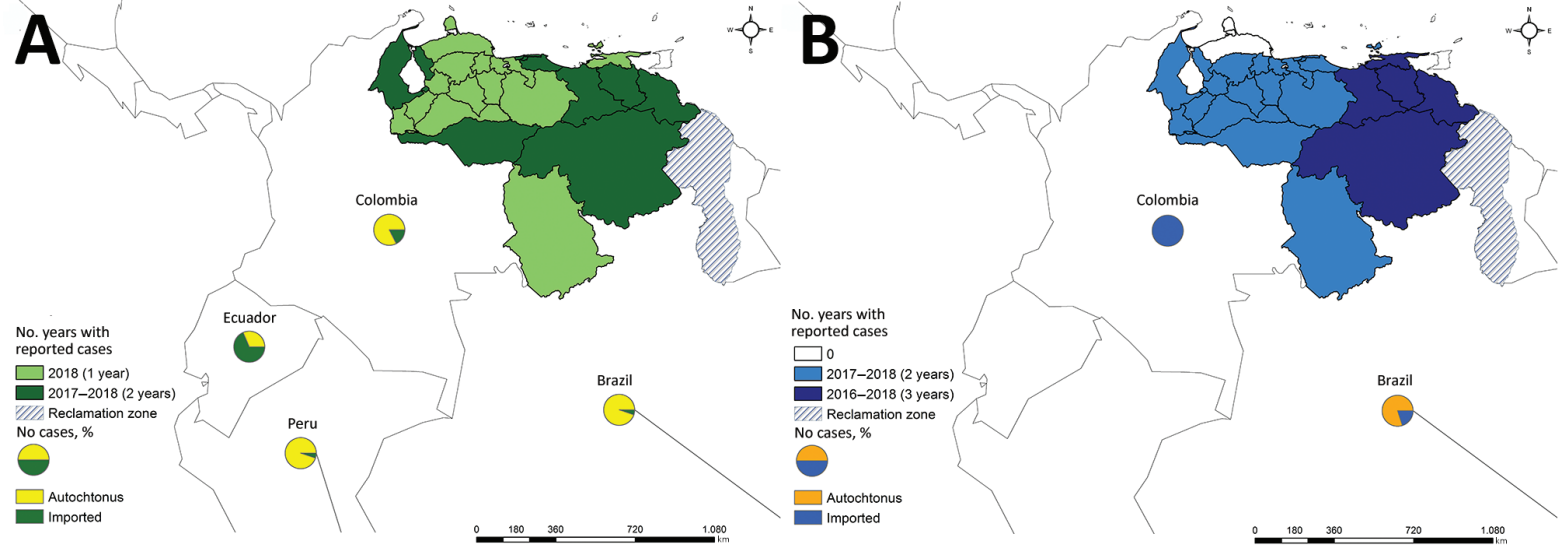
States affected by (A) measles and (B) diphtheria (blue), Venezuela, 2017–2018. Circles indicate neighboring countries reporting imported and autochthonous cases of these 2 diseases. Reclamation zone is a territory under dispute between Guyana and Venezuela.

Measles now affects multiple states in Venezuela, and a disproportionate number of cases occur among indigenous populations located in the vast forestlands of the southern region. Measles cases were first reported in epidemiologic week 26 of 2017 in the southern, mineral-rich state of Bolívar; 82% of the cases were detected in the municipality of Caroní (*18*). From week 26 of 2017 through week 43 of 2018, a total of 7,524 suspected cases were reported, of which 6,252 have been confirmed (727 in 2017 and 5,525 in 2018); 75 deaths were attributed to measles, most of them in the Amazon and Orinoco River Delta regions of the country. The affected regions have widened to include other more populated states such as Apure, Anzoátegui, Delta Amacuro, the Capital District, Miranda, Monagas, Vargas, and Zulia (Figure 2, panel A) (*16*).

The circulation of measles in Venezuela was preceded by the progressive interruption of the national immunization program since the year 2010, along with the dismantling of the primary healthcare infrastructure. The national coverage rate for the second dose of the measles vaccine was estimated at 52% according to the last reports from the Venezuela Ministry of Health (*19*,*20*). This estimate ranks Venezuela toward the bottom of vaccination coverage in the region (*20*). Current estimates indicate that measles vaccination coverage in the Venezuelan Amazon region has decreased in all municipalities (Alto Orinoco, 40%; Atabapo 18, 6%; Atures 66, 6%; Autana 35, 5%; Manapiare 30, 5%; Maroa 5, 2%; and Rio Negro 41, 7%) (*21*), notably affecting the Venezuela–Brazil border, where the current measles outbreak threatens to decimate the ancestral aboriginal Yanomami people.

The massive job losses that followed the economic crisis and the dismantling of private industry has meant that most persons in Venezuela have been forced to rely on informal jobs, such as illegal mining. Illegal mining camps, which attract migrant workers from communities throughout Venezuela and from neighboring countries and aboriginal settlements, provide a setting for measles transmission within the camps themselves and for its further spread once the mine workers return to their home communities. Cross-border mobility, migration, and illegal mining activities (*21*), in which native and indigenous persons are increasingly involved, have been proposed as the probable source of the proliferation of measles in remote areas inhabited by unvaccinated persons (*21*). Examples of vulnerable aboriginal settlements include those located at the Imataca Forest Reserve; the Paragua, Caura, and Caroní Rivers; Cerro Guanay Natural Monument; and most important, the Canaima and Cerro Yapakana National Parks (*22*). Recent reports from the nongovernmental organizations Survival International and Wataniba indicate that ≈100 cases of measles have occurred among members of the isolated and vulnerable Yanomami populations in the Venezuela–Brazil border region (*23*). Deaths attributed to measles have already occurred, according to Horonami (a Yanomami nongovernment organization), and high morbidity and mortality rates are expected because these populations are immunologically naive (*24*,*25*). Measles is presumed to have entered the Yanomami communities from Brazil after imported cases from Venezuela brought the disease to border populations of Brazil, before spreading back to Yanomami communities in Venezuela (*11*,*25*). Further spread of this epidemic wave could devastate the Yanomami people living in the Orinoco highlands of the Amazon, given that humanitarian aid to affected sites is limited or hard to deliver because of the seminomadic characteristics of these indigenous populations and the remoteness of the Yanomami territory.

Other indigenous populations in Venezuela who are particularly vulnerable to the extension of the measles outbreak include the Warao, located in the Orinoco River Delta. During 2018, ≈100 measles-related deaths occurred among indigenous infants and children in the municipality of Tucupita in the Orinoco River Delta (*24*). Reports from the municipalities of Pedernales and Antonio Diaz in the same area indicate that the death toll has increased, with 102 additional cases to date (*24*). Cases have spread rapidly from this region to the neighboring municipality of Barrancas del Orinoco (Venezuela) (*25*) and to the border state of Roraima (Brazil).

Again, emigration from Venezuela has substantially affected neighboring Brazil and Colombia, as well as other countries in the region, such as Ecuador (*26*). Measles cases imported from Venezuela have been reported in all 3 nations (Figure 1, panels A, B) Thus far, Brazil has reported most of the imported measles cases, mainly from the state of Bolivar in Venezuela), where a major measles outbreak is taking place (*27*). Not surprisingly, Brazil has the region’s second highest measles burden, with 2,801 confirmed cases reported through epidemiologic week 44 of 2018 (Appendix) in the neighboring states of Roraima (345 cases) and Amazonas (2,357 confirmed cases and 7,425 suspected cases) (Figure 2, panel A) (*10*,*24*). The link to the Venezuela outbreak was established by the isolation and identification of genotype D8 in the first cases of the outbreak in Brazil in February 2018, as confirmed by the Oswaldo Cruz Foundation and unequivocally recognized by PAHO (*18*,*24*). In Brazil, the locations principally affected are Boa Vista, Paracaima, Cantá, Rorainopolis, Uramuta, and Manaus (*24*). These states have received a massive influx of migrants from Venezuela (*24*). Isolated and import-related cases were also identified in the states of São Paulo (3 cases), Rio Grande do Sul (43 cases), Pará (23 cases), Pernambuco (4 cases), and Rondônia (2 cases), as well as in Rio de Janeiro (19 cases), Sergipe (4 cases), and Distrito Federal (1 case) (*24*). In addition, the Brazilian Ministry of Health has reported 14 measles deaths through the end of October 2018 in the states of Roraima (4 cases), Amazonas (8 cases), and Pará (2 cases) (*24*). Of all cases reported by Brazil’s Ministry of Health in the state of Roraima through May 2018, a total of 68% corresponded to refugees from Venezuela, and 52.7% were in Warao Amerindians (*24*). Although Brazil has a free vaccination program with high coverage levels, a recent decrease in polio and measles, mumps, and rubella vaccination rates, linked to local antivaccine movements (*28*,*29*) and government underfunding of the healthcare system (*30*), are worrisome and might worsen the current measles outbreak (*28*,*29*).

The Colombia Ministry of Health has also reported 25 measles cases imported from Venezuela (Figure 2, panel A) (*16*), again confirmed by isolation and molecular identification of genotype D8 in the initial cases (*18*,*31*). Most cases were reported from bordering states that received a considerable migratory influx in 2018 and that also had received the most numerous groups comprising the 600,000 immigrants from Venezuela in 2017 (*26*). Moreover, many residents of Venezuela transit through Colombia to Ecuador, where, by epidemiologic week 44 of 2018, a total of 13 of the 600 suspected cases imported from Venezuela had been confirmed as measles by the Ecuador Ministry of Health (Figure 2, panel A) (*8*,*10*). The index case-patient proceeded from Venezuela through the border with Colombia, where ≈286,000 persons have crossed through the Rumichaca international bridge. As of epidemiologic week 42 of 2018, the Peru Ministry of Health had also reported 2 cases imported from Venezuela (Figure 2, panel A) (*16*).

The reestablishment of measles transmission because of continuous circulation of either indigenous or imported measles virus for >12 months (*32*) has threatened the Americas with losing its certification as a measles-free region. On August 24, 2018, PAHO recognized the reestablishment of endemic transmission of measles in Venezuela, thereby suggesting that the Americas was unlikely to remain a certified measles-free region (*33*,*34*).

## Diphtheria

Diphtheria, a childhood vaccine-preventable disease, had not been reported in Venezuela during the 24 years before 2016. However, during July–November 2016, a total of 183 suspected diphtheria cases were reported by 16 of the 24 federal entities in Venezuela (Figure 2, panel B) (*34*), alerting PAHO and WHO to the reappearance of this disease (*35*). Since 2016, a total of 2,170 cases have been reported to date (324 in 2016, 1,040 in 2017, and 806 cases in 2018 [through October 29]). Of the 2,170 cases, 1,249 were laboratory confirmed; 287 deaths were reported (17 in 2016, 103 in 2017, and 167 in 2018), yielding a case-fatality rate of 22% (*34*). For 2018, a total of 838 diphtheria cases have been reported in the Americas as of epidemiologic week 41 of 2018 (*34*). The other 3 countries are Haiti (80 cases), Brazil (6 cases) (*36*), and Colombia (8 cases). However, 3 of 8 cases in Colombia were imported cases from Venezuela (*34*).

As in the case of measles, low local vaccination coverage rates have left Venezuela susceptible to the resurgence of diphtheria. By 2016, national coverage with 3 doses of diphtheria-tetanus-pertussis (DTP) vaccine (DTP3) was ≈84%, and reported coverage with 4 doses of DTP barely reached 60% (*37*). PAHO estimated coverage rates in 2017 of 66% for DTP3 and 38% for 4 doses of DTP (*38*). Recent unofficial data suggest that for 2018, national DTP3 coverage might not even reach 50% (*39*), resulting in an important increase in the number of susceptible persons, of whom ≈3 million are children. Achieving adequate vaccination rates is even more difficult in remote areas, where geographic isolation and ongoing conflicts between armed gangs, guerrilla forces, and military personnel hamper adequate access to rural communities. The southern areas of the Amazon basin, such as Bolívar state, Amazonas state, and the Orinoco River Delta, are particularly at risk because of low pentavalent DTP–*Haemophilus influenzae* type b–hepatitis B vaccination coverage; reported coverage rates are 50% for Bolívar, 37% for Amazonas, and 24% for the Orinoco River Delta, according to data from the Venezuela Ministry of Health (*37*,*40*).

Although the first cases of diphtheria in Venezuela were reported in the southern state of Bolivar and were attributed initially to crowding and unhealthy conditions in illegal mining camps, the disease has spread rapidly throughout the country, reaching epidemic proportions (*39*). Current data indicate that the number of reported cases likely underestimates the actual magnitude of the outbreak. Reports on the occurrence of diphtheria in isolated Amerindian communities, such as the Pemón and Kariña aboriginal populations, illustrate the geographic reach of the disease.

Diphtheria has now spread to Brazil and Colombia (Figure 2, panel B). In February 2018, PAHO–WHO issued an update on diphtheria in the Americas, highlighting the occurrence of exported cases from Venezuela to Brazil and Colombia (*41*). In Brazil, a fatal case imported from Venezuela was recorded in the state of Roraima, and in Colombia, another fatal case was reported in the Department of La Guajira (Figure 2, panel B). These cases highlight the vulnerability of the bordering states to the outbreak (*41*). The lack of infrastructure to receive massive numbers of forced migrants and the associated problems with poor access to essential health and sanitation services are potential facilitators for disease emergence and transmission (*41*). During epidemiologic week 8 of 2018, PAHO urged health authorities to intensify epidemiologic surveillance, case detection, medical care, and vaccination (*39*). Since that week, PAHO has confirmed diphtheria cases in Brazil, Colombia, and Venezuela (Figure 2, panel B) (*41*).

## Polio

In 1971, poliomyelitis became the second vaccine-preventable disease (after smallpox) to be eliminated from the Americas, later followed by measles and rubella (*42*,*43*). Venezuela’s devastated healthcare infrastructure has halted many of its immunization programs. Estimates indicate that vaccination coverage against polio has dropped below minimum recommended levels (80%); coverage with the third dose of polio vaccine slipped from 87% in 2015 to <79% in 2017 (*7*), thus establishing the conditions for the potential emergence of vaccine-derived polioviruses (VDPVs). Historically, low vaccination coverage in conflict-affected countries has played an important role in the reemergence of poliomyelitis because of circulating VDPVs (cVDPVs). Examples include Laos, where an outbreak attributable to cVDPV type 1 cases occurred during 2015–2016; Nigeria and Pakistan, which reported cVDPV type 2 cases throughout 2016; Syria and the Democratic Republic of the Congo, where cVDVP type 2 was present during 2017–2018; and Mogadishu, Somalia (2017) and Kenya (2018), where cVDPV type 2 was isolated from environmental samples (*44*).

The current reality in Venezuela is a conflux of plummeting vaccination coverages and ongoing outbreaks of other vaccine-preventable diseases. Combined with the weakening of surveillance programs, forced migrations, and a prolonged political, economic, and food crisis without foreseeable resolution, these factors have set the stage for potential reemergence of poliomyelitis.

## Addressing the Vaccine-Preventable Disease Crisis in Venezuela

During the past decade, crises in Africa and the Middle East have provided numerous examples of the consequences for failure of the control of vaccine-preventable diseases when healthcare delivery is disrupted by political turmoil; these areas have also provided paradigms of successful intervention measures. In Syria, the destruction of healthcare and sanitation infrastructure resulted in the reappearance of polio 15 years after its eradication from that country. At the same time, the number of measles cases expanded to >7,000 confirmed cases nationwide and extended into neighboring countries with higher vaccination coverage (*45*). Similarly, disruption of immunization programs during the recent political unrest in Yemen led to a measles outbreak with >4,300 cases and a high death rate (*46*); the number of cases totaled 7,285, according to the most recent data from WHO (*47*). In both Syria and Yemen, the acknowledgment by WHO of the severity of the crises and assistance marshaling the resources required to mount massive immunization campaigns enabled substantial progress toward containment, although the inability to consistently sustain immunization activities has precluded ideal disease control and the prevention of potential new outbreaks. Subsequently, WHO codified and published an evidence-based approach for vaccination in humanitarian crises that incorporates a framework for decision-making (*48*). The ongoing diphtheria and measles epidemics in Venezuela and spillover into neighboring countries evoke the reemergence of vaccine-preventable diseases observed in Syria and Yemen and the consequent threat to regional, and potentially global, public health.

The Americas region has been free of wild poliovirus circulation for nearly 3 decades, mainly because of successful immunization programs and vaccination campaigns in high-risk regions. Without doubt, these efforts halted the final chains of transmission and provided strong herd immunity. Similarly, in 2007, Venezuela was able to successfully implement a mass vaccination program that arrested the circulation of measles and ended the 2006 outbreak. Today, however, the crisis in Venezuela has enabled vaccine-preventable diseases such as diphtheria and measles to reemerge (*34*). The weakening of Venezuela’s public health services has led to a breakdown of epidemiologic surveillance systems along with an interruption of the national immunization program, resulting in the decay of infection control practices. In addition, the ongoing massive internal and external exodus of Venezuela residents has become the amplifying factor of these outbreaks beyond Venezuela’s borders (*8*,*24*,*26*,*29*,*49*).

The Executive Committee of PAHO, at its 162nd session held on June 20, 2018, presented the following document as a point on its agenda: “PAHO Response to Maintain an Effective Agenda for Technical Cooperation in Venezuela and in Neighboring Member States” (*49*). The document contributes, albeit somewhat late, to addressing the lack of information and to correct misinformation, as well as to refute the government of Venezuela’s official denial of the serious problems afflicting the country. Likewise, the document recognizes the lack of official information but fails to indicate as a priority the resumption of the publication of the Venezuelan Ministry of Health’s Weekly Epidemiologic Bulletins and relevant technical documents from the Venezuela Ministry of Health. We believe that reporting health statistics is an unavoidable obligation of the state to its inhabitants and cannot be substituted by regional and international bulletins or alerts from other countries.

## Recommendations

According to the evaluation approach recommended by WHO, the risk level of the ongoing outbreaks in Venezuela is high. A correspondingly strong response is needed to curtail the expanse of these epidemics. We propose the following measures.

- Global and hemispheric health authorities should urge the Venezuela government to allow the establishment of a humanitarian channel to provide immediate relief efforts addressing extreme food and medicine shortages.
- Epidemiologic surveillance programs, early reporting, and rapid response systems should be restored immediately. Strengthening of infection control practices in healthcare facilities should be implemented with the aid of international agencies while ensuring public health neutrality.
- Emergency relief operations should be put into effect across borders along with authorities in Colombia and Brazil to ameliorate the effects of massive migration by implementation of early nutritional and immunization interventions.
- International agencies should support regional efforts in neighboring countries to promote simultaneous massive vaccination campaigns and vaccination of all refugees from Venezuela arriving in host community populations.
- Adequate supplies for mass vaccination and routine immunization should be ensured, and additional adjunct supplies (e.g., diphtheria antitoxin) should be stockpiled to assist in the establishment of standard treatment protocols and epidemic rapid response measures. These methods are crucial for healthcare delivery and mass vaccination catch-up campaigns to head off the resurgence of vaccine-preventable diseases in Venezuela.
- In areas with low vaccination coverage, improving surveillance for early case detection and increasing vaccination coverage in high-risk age groups should be mandatory. Furthermore, Venezuela is in urgent need to reconstruct its devastated healthcare system, secure sustainable food and medication access, and reinstall proper sanitation policies to reduce the burden of diseases.

On September 27, 2018, the United Nations Human Rights Council adopted a resolution on Venezuela signaling the gravity of the human rights situation and the growing concern by governments worldwide about the country’s humanitarian crisis, including aspects such as malnutrition and the upsurge of preventable diseases (*50*). PAHO–WHO faces an enormous challenge in attending, without interference, to the complex emergency that affects Venezuela. Emergency funds must be released to acquire medicines, vaccines, laboratory reagents, and other supplies for health programs. As Venezuela rapidly becomes a regional nidus for the emergence of vaccine-preventable diseases, it must take decisive action now alongside regional and national partners to target this emerging regional crisis.

Appendix Additional information regarding the regional public health threat in the Americas caused by resurgence of vaccine-preventable diseases in Venezuela.
