# Supplementary material for: Resurgence of Vaccine-Preventable Diseases in Venezuela as a Regional Public Health Threat in the Americas
Source: Emerg Infect Dis. 2019 Apr;25(4):625–32. doi: 10.3201/eid2504.181305 (PMC6433037; doi:10.3201/eid2504.181305)
Supplement: Appendix — Additional information regarding the regional public health threat in the Americas caused by resurgence of vaccine-preventable diseases in Venezuela. [file 18-1305-Techapp-s1.pdf]

Article DOI: https://doi.org/10.3201/eid2504.181305

Resurgence of Vaccine-Preventable Diseases in Venezuela as a Regional Public Health Threat in the Americas

Appendix

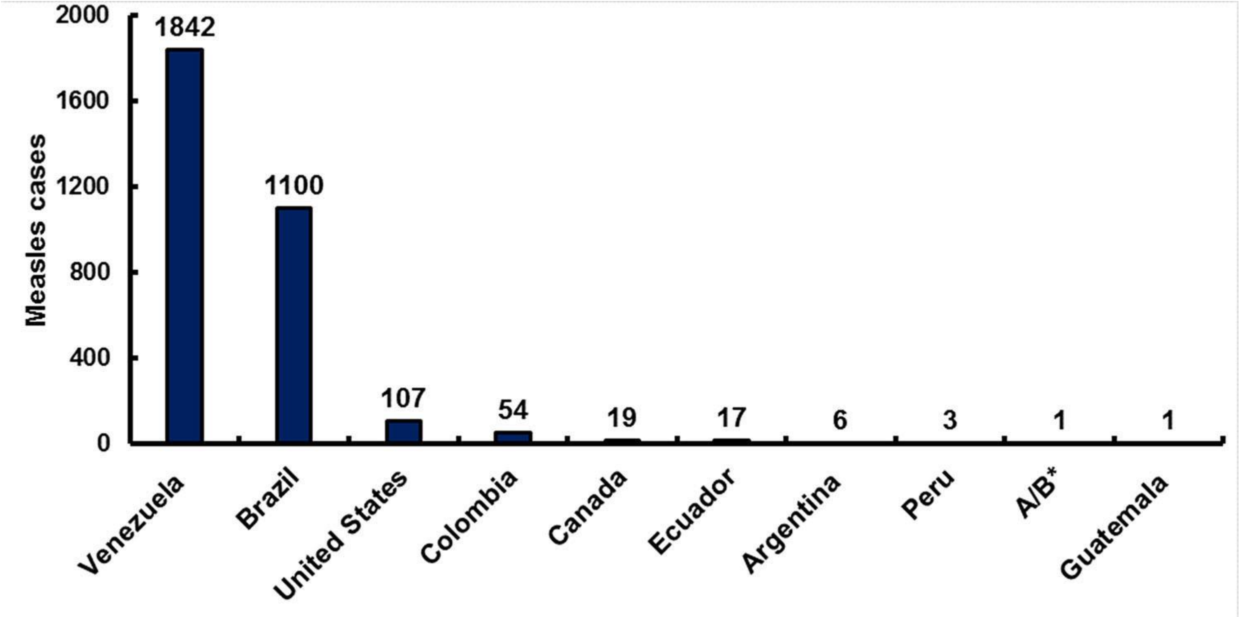

Appendix Figure. Number of confirmed measles cases, the Americas, epidemiologic weeks 1–44, 2018.
